# Supplementary material for: Treatment of Lumbar Degenerative Disease with a Novel Interlaminar Screw Elastic Spacer Technique: A Finite Element Analysis
Source: Bioengineering (Basel). 2023 Oct 16;10(10):1204. doi: 10.3390/bioengineering10101204 (PMC10604319; doi:10.3390/bioengineering10101204)
Supplement: Supplementary file 1 [file bioengineering-10-01204-s001.zip › bioengineering-2617823-supplementary.pdf]

Figure S1

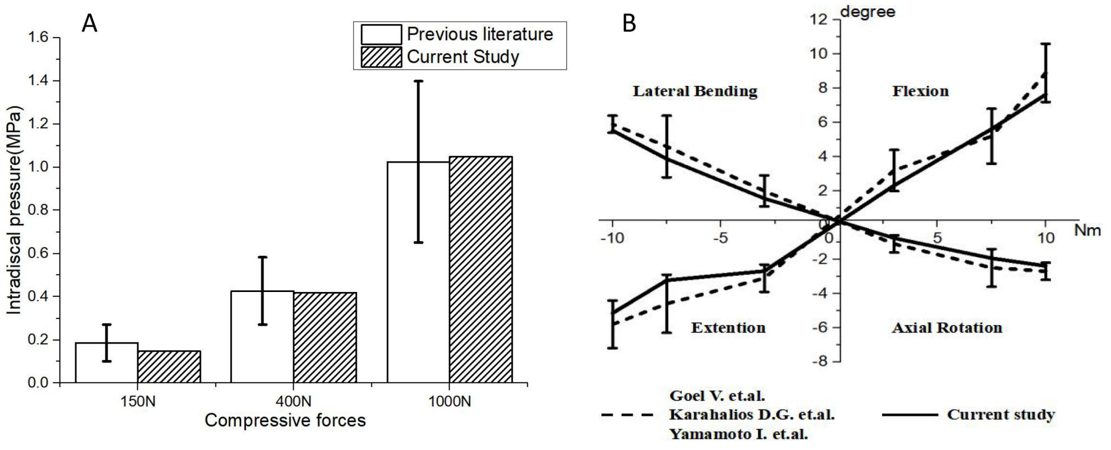

Validation of L4-L5 spinal finite element model. (A) Comparison of intervertebral disc stress at the L4-L5 level and the in vitro experimental data from Dreischarf et. al. and Brinckmann et. al., (B) ROM of L4-L5 lumbar spine.
